# ‘Omics Approaches to Explore the Breast Cancer Landscape

**DOI:** 10.3389/fcell.2019.00395

**Published:** 2020-01-22

**Authors:** Joseph Parsons, Chiara Francavilla

**Affiliations:** ^1^Division of Molecular and Cellular Function, School of Biological Sciences, Faculty of Biology Medicine and Health, The University of Manchester, Manchester, United Kingdom; ^2^Division of Cancer Sciences, School of Medical Sciences, Faculty of Biology Medicine and Health, The University of Manchester, Manchester, United Kingdom

**Keywords:** breast cancer, system biology, proteomics, transcriptomics, genomics, organoids, PDX

## Abstract

Breast cancer incidence is increasing worldwide with more than 600,000 deaths reported in 2018 alone. In current practice treatment options for breast cancer patients consists of surgery, chemotherapy, radiotherapy or targeting of classical markers of breast cancer subtype: estrogen receptor (ER) and HER2. However, these treatments fail to prevent recurrence and metastasis. Improved understanding of breast cancer and metastasis biology will help uncover novel biomarkers and therapeutic opportunities to improve patient stratification and treatment. We will first provide an overview of current methods and models used to study breast cancer biology, focusing on 2D and 3D cell culture, including organoids, and on *in vivo* models such as the MMTV mouse model and patient-derived xenografts (PDX). Next, genomic, transcriptomic, and proteomic approaches and their integration will be considered in the context of breast cancer susceptibility, breast cancer drivers, and therapeutic response and resistance to treatment. Finally, we will discuss how ‘Omics datasets in combination with traditional breast cancer models are useful for generating insights into breast cancer biology, for suggesting individual treatments in precision oncology, and for creating data repositories to undergo further meta-analysis. System biology has the potential to catalyze the next great leap forward in treatment options for breast cancer patients.

## Breast Cancer – Where Are We?

Breast cancer is the leading cause of cancer-related deaths in women worldwide (Bray et al., 2018). It is a heterogeneous disease (Nik-Zainal et al., 2016), commonly separated into Luminal A (LumA), Luminal B (LumB), epidermal growth factor receptor ERBB2/HER2-overexpressing (HER2+), basal epithelial-like (BL) based on gene expression profiles (Sørlie et al., 2001). Breast cancer is currently treated with surgery, radiotherapy, cytotoxic chemotherapy and/or targeted therapies to eradicate viable cancer cells (Fisher et al., 2002).

LumA and LumB breast cancers are both estrogen receptor (ER)-positive (Sørlie et al., 2001). Deregulated ER signaling is associated with cancer hallmarks (Hanahan and Weinberg, 2011). For instance, ER target genes like cyclin-dependent kinase (CDK) 1 or the kinase Src promote cell proliferation, invasion and epithelial–mesenchymal transition (EMT) (Stender et al., 2007; Saha Roy and Vadlamudi, 2012). LumB cancers have high expression of the proliferation marker Ki67, which correlates with increased risk of developing distant metastases (Colzani et al., 2014), and reduced expression of the progesterone receptor (PR) (Cho, 2016), which shifts gene expression toward more tumorigenic genes (Mohammed et al., 2015). LumA and LumB tumors are treated using ER antagonists (e.g., tamoxifen), aromatase inhibitors and selective estrogen receptor degraders (e.g., fulvestrant). However, therapeutic resistance may arise through loss of ER expression, mutations in ER or overexpression of alternative breast cancer-driving pathways such as ERBB1/EGFR (Garcia-Becerra et al., 2012; Clarke et al., 2015; Ma et al., 2015). To overcome resistance to traditional ER antagonists targeted therapies against phosphoinositide 3-kinases (PI3K), mammalian target of rapamycin (mTOR), and CDK4/6 have recently been proven beneficial in the clinical setting (Beaver and Park, 2012; Kornblum et al., 2018; Pernas et al., 2018).

HER2 + breast cancers overexpress ERBB2/HER2 (Iqbal and Iqbal, 2014) which promotes proliferation by regulating CDKs and Cyclins (Timms et al., 2002). Additionally, HER2 dimerization with EGFR induces activation of mitogen-activated protein kinase (MAPK), c-Jun N-terminal kinases (JNK), and phosphoinositide phospholipase C (PLCγ) signaling pathways resulting in increased cell proliferation, migration and apoptosis resistance (Masuda et al., 2012). HER2 + breast cancers are treated with targeted agents such as trastuzumab, pertuzumab, and neratinib. Trastuzumab is an antibody which inhibits HER2 dimerization, promotes natural killer cell recruitment to tumors and stimulates ubiquitin-dependent HER2 degradation (Vu and Claret, 2012; McCann and Hurvitz, 2018; Schmid et al., 2018; Vikas et al., 2018). Therapeutic resistance to trastuzumab occurs via HER2 dimerization with other ERBB family members or constitutive HER2 activation (Vu and Claret, 2012).

BL breast cancers do not generally express ER, PR or HER2 (Milioli et al., 2017), like triple negative breast cancers (TNBCs) (Lehmann et al., 2016). BLs are highly heterogeneous and include basal-like1-2, claudin-low, and immunomodulatory subgroups (Garrido-Castro et al., 2019). BLs have a highly proliferative and invasive phenotype with high risk of relapse in early breast cancer (Fallahpour et al., 2017). BLs are typically treated by chemotherapy and radiotherapy (Wahba and El-Hadaad, 2015) although recent advances have led to novel treatment opportunities for BL cancer patients. For instance, immunomodulatory BLs can be treated with immune checkpoint programed cell death protein 1 (PD-1) and poly (ADP-ribose) polymerase (PARP) inhibitors (McCann and Hurvitz, 2018; Schmid et al., 2018; Vikas et al., 2018).

Two major challenges in breast cancer treatment are therapeutic resistance and the formation of metastasis to secondary sites (lung, bone, lymph nodes, brain, and liver) inevitably leading to patient mortality (Minn et al., 2005). As 10 year survival for metastatic breast cancer patients remains below 5% (Kontani et al., 2014) and response to targeted therapies varies from 15 to 40% for all subtypes (Bartsch et al., 2007; Haque and Desai, 2019) the need for novel therapeutic options for breast cancer patients remains a priority.

Here, we will describe several models that have contributed to knowledge of breast cancer biology and the repertoire of currently available therapeutic targets. Thereafter, we will introduce system biology-based approaches and finally discuss how their integration with traditional models is revolutionizing breast cancer translational research.

## Models to Study Breast Cancer

### Cell Lines

**FIGURE 1:**
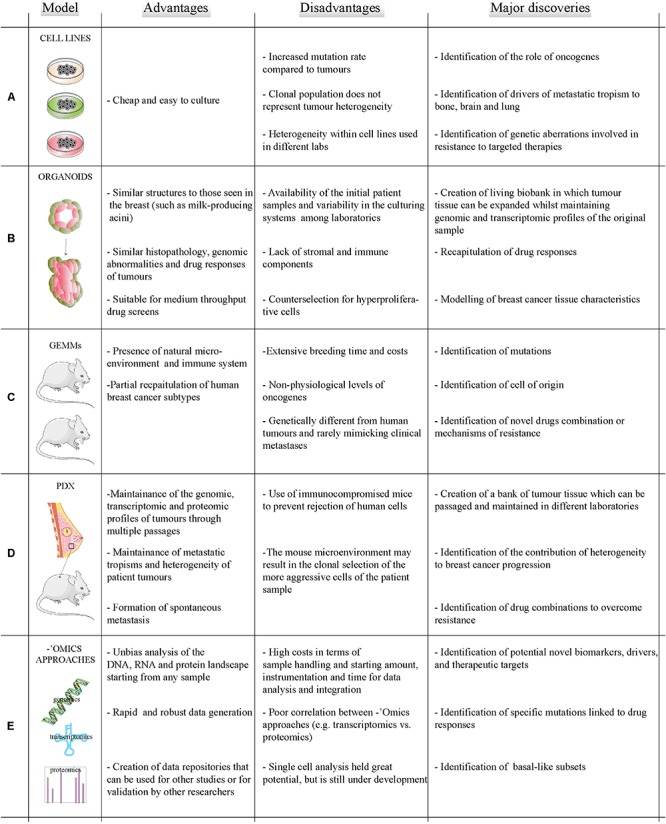
Models and methods to study breast cancer. Summary of the advantages (left column) and disadvantages (middle column) of existing breast cancer models **(A–D)** and ‘omics technologies **(E)** to study breast cancer. Right column reports a brief summary of how different methods and models have contributed to major discoveries in the field of breast cancer.

Breast cancer has been traditionally studied using immortalized cell lines derived from patient samples (Holliday and Speirs, 2011) which are easy and inexpensive to grow. These cell lines express biomarkers of the different molecular subtypes of breast cancer (Dai et al., 2017) and recapitulate some parent tumor characteristics including drug responses (Holliday and Speirs, 2011) and transcriptomic profiles (Neve et al., 2006). Cell lines have enabled major discoveries in breast cancer research, such as the identification of oncogenes (Elenbaas et al., 2001) and drivers of metastatic tropism (Minn et al., 2005). However, breast cancer cell lines have increased gene copy number variations compared to primary tumors (Larramendy et al., 2000), lack the *in vivo* microenvironment (Vincent et al., 2015), and do not maintain primary tumor heterogeneity (Dai et al., 2017; Liu et al., 2019) (Figure 1A).

### Organoids

Organoids are three dimensional (3D) cell cultures which mimic healthy tissues and cancer lesions (Xu et al., 2018). Organoids are usually grown in matrices such as Matrigel^TM^, collagen or peptide hydrogels which aim to recapitulate the breast microenvironment (Djomehri et al., 2019). The group of Mina Bissel in the ‘80s began to investigate how organoids were a better model for studying breast tissue compared to 2D cell culture (Weaver et al., 1995). More recently, primary and metastatic organoids have been developed which accurately recapitulate parent tumor characteristics including histopathology, genomic abnormalities and drug responses (Sachs et al., 2018). Organoids are easy to modify, can be propagated for up to 3 months (Fatehullah et al., 2016), and allow drug screening (Dutta et al., 2017). Recently, the issue of availability of primary patient samples for laboratories without access to biobanks has been solved by the creation of living biobanks of frozen organoids (Dutta et al., 2017). Organoids can be used as models to study different breast cancer subtypes and to identify potential novel therapeutic targets. Organoid are better models than 2D cultures to analyze drug response due to a more representative microenvironment and selection for stem-like cells, like those responsible for metastatic initiation (Velasco-Velazquez et al., 2011; Imamura et al., 2015). Despite these promising characteristics for breast cancer translational research, organoids lack components of the *in vivo* microenvironment and may suffer for counterselection of hyperproliferative cells (Fujii et al., 2016; Weeber et al., 2017) (Figure 1B).

### Genetically Engineered Mouse Models (GEMMs) and Syngeneic Mouse Models (SMMs)

*In vivo* modeling of breast cancers generally entails inducing oncogene expression (e.g., *Erbb2*) or knocking out a tumor suppressor gene (e.g., *p53*) in mice. Examples include the mouse mammary tumor virus (MMTV) promoter-driven or the 4T1-based SMMs (Holen et al., 2017). GEMMs include a natural (mouse) microenvironment and immune system, and partially mimic all human subtypes save luminal cancers (Pfefferle et al., 2013; Holen et al., 2017). However, GEMMs involve extensive costs and breeding time, often express supra-physiological levels of the transgene, and can be genetically different compared to their human counterpart (Pfefferle et al., 2013). Only 16 of the 30 most commonly mutated genes in human breast cancers were found to be mutated in a panel of metastatic GEMMs and SMMs (Yang et al., 2017). Although SMMs have higher mutational burden in metastases than in primaries like human breast cancers (Yang et al., 2017; Yates et al., 2017), GEMMs and SMMs rarely mimic clinical metastasis (Holen et al., 2017). In spite of these pitfalls, GEMMS have been instrumental in generating insights into breast cancer biology – e.g., determining that BRCA1 mutant tumors derive from luminal progenitor rather than basal cells (Molyneux et al., 2010) and in testing novel drugs combinations (Jaspers et al., 2013) (Figure 1C).

### Patient-Derived Xenografts (PDXs)

Patient-derived xenografts (PDXs), which involve injection of human cancer cells either orthopically in the mouse mammary fat pad or subcutaneously into immunocompromised mice, provide an *in vivo* alternative to GEMMs (Hidalgo et al., 2014; Holen et al., 2017). They have helped address clinically relevant questions including the contribution of heterogeneity to, and the mechanism of, drug resistance (Byrne et al., 2017). PDXs can be passaged in different mice allowing expansion of patient tissue whilst still maintaining ‘omics profiles of the patient tumor; and they spontaneously metastasize (DeRose et al., 2011; Dobrolecki et al., 2016). Drawbacks for the use of PDXs include the selection of more aggressive cells within the patient sample and the use of immunocompromised mice to prevent tumor rejection. Developing mice with humanized immune systems can help to address this problem (Hasgur et al., 2016), as recently shown for a metastasis model (Rosato et al., 2018) (Figure 1D).

In conclusion, choosing the correct model to study breast cancer depends on several factors including the biomedical question, sample availability, costs, etc. (Figure 1). We envision that future interdisciplinary research will be based on a combination of different models to identify and validate new therapeutic targets for breast cancer treatment with the advent of next generation sequencing and more robust instrumentation, ‘omics approaches, like genomics and proteomics, are becoming more accessible and are increasing the information that can be obtained from breast cancer models. Thus, ‘omics approaches applied to the combination of different models will provide molecular information on a global scale and will identify novel targets.

## System Biology Approaches to Study Breast Cancer

System biology based on ‘omics approaches and network science are becoming popular in cancer research (Manem et al., 2018), despite high costs in terms of sample handling, instrumentation, and time for data analysis. Integrating ‘omics approaches allows the unbiased analysis of the whole genome, transcriptome, proteome, or metabolome starting from different types of samples (Figure 1E and Table 1).

**TABLE 1:** A selection of single- and multi-‘omics-based breast cancer studies that have contributed to major discoveries in the field of breast cancer research where method strengths and weakness are reported.

**Study**	**Topic area**	**‘Omics approaches**				**Method strengths**	**Method weaknesses**	**Major discoveries**
		**G**	**T**	**P**	**M**			
Nik-Zainal et al., 2016	Novel Breast Cancer Drivers	×				The whole genome sequence can be determined relatively cheaply in less than a week	Sequences must be read many times to account for inaccuracies in sequencing analyzers	Five novel cancer genes were identified. A total of 93 genes were suggested to contain breast cancer driver mutations
Playdon et al., 2017	Breast Cancer Risk				×	This technique is dependent on serum samples which are far easier to obtain than biopsies needed for other ‘omics techniques	Controlling patient diet is very difficult	Three metabolites were found to be associated with increased breast cancer risk
Varešlija et al., 2018	Novel Therapeutic Targets	×	×			Combining DNA and RNA sequencing allows mutations to be connected to chromatin remodeling and gene expression	RNA integrity is compromised by the process of formalin fixing due to cross-link formation	RET and HER2 were found to be potential therapeutic targets for breast cancer brain metastases
Huang K. L. et al., 2017	Novel Therapeutic Targets	×	×	×		Proteomic isobaric labeling methods allow multiple samples to undergo relative quantification reducing variability	Large amounts of starting protein is required for phospho- proteomics. Also proteomic labeling reagents are very expensive	Novel therapeutic targets previously undiscovered at the genomic, transcriptomic or proteomic level were identified at the level of the phosphoproteome in PDX models
Massard et al., 2017	Informing Clinical Therapeutic Decisions	×	×			When tumor cell population is low in a biopsy, targeted sequencing of known cancer genes can still be used to search for actionable targets without having to purify the epithelial population	Extensive analysis is required to determine if a mutation is actionable. Also biopsies are often sent to pathologists before freezing so the molecular profile may be changed by the time the tissue is frozen	The treatment of 199 patients was based on an actionable genomic alteration which was found using DNA and RNA sequencing In 33% of patients. progression-free survival was significantly increased and in 11% there was objective response
Mertins et al., 2016	Breast Cancer Signaling	×		×		In situations where mutations produce unpredictable consequences, e.g., altering splice variants, proteogenomics can identify single amino acid variants and link these to mutations	Proteins which are missing in one or more replicates of a proteomic experiment are often excluded despite the fact the protein may have been present below the detection threshold	A number of highly phosphorylated kinases were identified that were not seen as potential therapeutic targets at the genomic level. Also the impact of mutations was traced to the signaling level to identify therapeutic targets, e.g., CETN3 loss was associated with EGFR upregulation. highlighting how this loss could be druggable
Johansson et al., 2019	Breast Cancer Subtypes	×	×	×	×	Integrating ‘omics technologies allowed the mRNA- based subtypes to be expanded to a more clinically useful resource	Tumors are heterogenous and so ‘omics data from one part of a biopsy may not be representative of the whole tumor	Breast cancer subtypes (Sørlie et al., 2001) were validated at a multi-omic level. Basal-like tumors were separated into two clusters that could inform therapeutic decisions

*G, genomics; T, transcriptomics; P, proteomics; M, metabolomics.*

### Genomics

Next generation sequencing (NGS) allows rapid and relatively inexpensive DNA sequencing covering the whole genome (Park and Kim, 2016). Genomic approaches helped redefine breast cancer subtypes (Cancer Genome Atlas Network, 2012), identify mutational landscapes (Stephens et al., 2012) or single nucleotide polymorphisms (SNPs) as a biomarker of breast cancer susceptibility (Michailidou et al., 2017) or therapeutic response (Kus et al., 2016). NGS has also facilitated the discovery of breast cancer driver mutations (Nik-Zainal et al., 2016), tumor heterogeneity (Yates et al., 2015) and novel therapeutic targets in metastatic disease (Bertucci et al., 2019). Finally, single-cell analysis allowed the study of breast cancer stem cells (Lawson et al., 2015). However, accurate genomic analysis requires large numbers of sequence reads which increases both time and cost.

These discoveries demonstrate the potential for genomics to transform breast cancer treatment (Hamdan et al., 2019). For instance, genomics helped identify patients for clinical trials (Curtis et al., 2012) or high risk individuals through mutation screening in breast cancer susceptibility (BRCA) 1–2 genes (Evans et al., 2008) and contributed to therapeutic decision making (Tsoutsou et al., 2017; Bergom et al., 2019). As an invaluable resource for researchers, the Catalogue of Somatic Mutations in Cancer (COSMIC) has compiled genomic data from breast cancer patient samples and correlated them to cellular functions and drug resistance (Forbes et al., 2017). Finally, genomic analysis for the early identification of tailored therapy for cancer patients has been made possible with the development of the Cancer Genome Atlas (TCGA)^1^. We envision that TCGA and COSMIC databases will revolutionize cancer patient diagnosis and treatment (Ashton-Prolla et al., 2015). This is already being realized in the MOSCATO trial where druggable genomic aberrations were identified and targeted in patients (Massard et al., 2017).

In addition, cell-free/circulating tumor DNA (cf/ctDNA) can be useful in monitoring clonal evolution and residual tumor presence following treatment (Buono et al., 2019). However, as ctDNA usually comprises 180–200 bp fragments from apoptotic cells, there are varying degrees of success in identifying useful biomarkers with high sensitivity (Sefrioui et al., 2015). Despite this, serial screening for mutations in ctDNA has allowed metastatic detection 8 months before clinical presentation (Garcia-Murillas et al., 2015).

Together with genomics, epigenomics (the study of DNA modifications and their impact) is also providing novel markers for breast cancer prognosis (Davalos et al., 2017) and for detection of metastasis (Legendre et al., 2015). Epigenomics has begun to illuminate the link between menopause and lifestyle factors with breast cancer risk and so may provide prognostic utility in future (Crujeiras et al., 2017).

### Transcriptomics

Transcriptomics uses microarrays, which quantify a set of predetermined sequences, and RNA sequencing (RNA-Seq), which uses high-throughput sequencing to capture all sequences to determine the quantity of a transcript (Lowe et al., 2017). These approaches have been used to classify breast cancer molecular subtypes in cell lines (Neve et al., 2006) and patient-derived samples (Wu et al., 2018; Jiang et al., 2019), to compare primary breast cancers and their metastases (Varešlija et al., 2018), and to visualize phenotypic features of breast cancer cells in 3D culture (Tirier et al., 2019). In addition, transcriptomics is allowing immune cell characterization in normal breast and tumor tissue (Chung et al., 2017; Azizi et al., 2018), potentially providing a mechanism to inform immunotherapeutic decisions.

As transcriptomics does not provide information on the expression, post-translational modifications (PTMs), or activation status of proteins it is less informative than proteomics for novel therapeutic target discovery. Recent advancements in single cell analysis may open a new era in breast cancer research to identify drivers, biomarkers, and novel therapeutic targets (Hong et al., 2019).

In the clinic, analysis of mRNA expression of gene subsets, involved in ER signaling, HER2 signaling, proliferation and invasion, is already used to predict relapse and determine whether patients would benefit from neoadjuvant chemotherapy (Vieira and Schmitt, 2018). Furthermore, as patients with elevated expression of a migratory mRNA signature had worse overall survival than those with a proliferative mRNA signature and so responded significantly better to chemotherapeutics that targeted the cytoskeleton (Nair et al., 2019) transcriptomics has the potential to inform chemotherapeutic decisions in future.

As patient tumor biopsies are typically formalin fixed and paraffin-embedded (FFPE), a preservation procedure that reduces RNA integrity (von Ahlfen et al., 2007), fresh frozen tissue collection should become the standard procedure for mRNA expression to inform clinical decisions.

### Proteomics

Proteomics studies the expressed proteome and its PTMs by mass spectrometry (MS), protein microarrays, and, more recently, mass cytometry. Advances in samples handling, instrumentation, and data analysis now provide unprecedented insights into the abundance and function of the (modified) proteome (Doll et al., 2019). Proteomics can assess tissue or blood samples, thus lending itself to clinical applications (Mardamshina and Geiger, 2017). For instance, specific serum biomarkers have been discovered by proteomic studies (Li et al., 2002; Raso et al., 2012), potentially providing an early diagnosis signature (Saadatmand et al., 2015). Correlation between RNA or gene copy number with protein expression is rather low (Mertins et al., 2016; Johansson et al., 2019) thus analyzing the patient proteome holds promise for identifying novel preventative or therapeutic targets not previously identified at the genomic or transcriptomic level. This idea is supported by the fact that currently used anti-breast cancer drugs predominantly act against proteins.

MS-based proteomics has been used to characterize cell lines (Huang F. K. et al., 2017), to reveal novel layers of breast cancer classification (Tyanova et al., 2016; Yanovich et al., 2018), and to identify proteins involved in drug resistance (Liu et al., 2006). Furthermore, phosphoproteomics that identify phosphorylated proteins (von Stechow et al., 2015) has been used to connect somatic mutations to signaling (proteogenomics) (Mertins et al., 2016), to identify kinases signatures in TNBC (Zagorac et al., 2018), and to map drug targets for personalized treatments (Pierobon et al., 2018). These discoveries have diagnostic and prognostic potential which is worth further exploring and implementing in the clinic when phosphoproteomics methods will become common practice.

An alternative to MS-based proteomics is provided by mass cytometry where single cells are probed with metal ion-labeled antibodies and then samples are analyzed by time-of-flight mass spectrometry (Leelatian et al., 2017). In breast cancer research this technology has been recently used to identify cell types and immune infiltrates within a tumor (Wagner et al., 2019). However, this method remains limited by antibody availability. Similarly to transcriptomics, phosphoproteomics is also limited by the availability of fresh frozen tissue as the phosphoproteome is substantially altered by FFPE preservation (Wakabayashi et al., 2014).

In conclusion, analyzing the proteome and phosphoproteome of patients at different breast cancer stages will help identify signatures for personalized treatments, ideally starting from liquid biopsies. In future proteomics may be used to follow the response to treatment by analyzing changes in patient proteome so to adapt the therapeutic plan.

### Metabolomics

Metabolomics is the system-wide identification of endogenous metabolites from bodily fluids in a targeted or unbiased manner (Silva et al., 2019). Metabolomics has been used to correlate changes in metabolism with proliferation rate in breast cancer cells (Jerby et al., 2012), to cluster tumor subtypes (Haukaas et al., 2016), to analyze the lipids content in breast cancer cells (Lisa et al., 2017), and to correlate nutrients with breast cancer risk (Playdon et al., 2017). More recently, this approach has begun paving the way for the identification of metabolic-state specific biomarkers for breast cancer diagnosis (Jasbi et al., 2019). Therefore, metabolomics will allow further insights into correlation between metabolism, epigenomic and proteomic alterations and breast cancer progression or treatment.

### Data Integration

The contribution of each aforementioned ‘omics technology to the understanding of breast cancer biology and to the discovery of novel targets or biomarkers has been substantial. Integrating these approaches is predicted to be even more powerful (Chakraborty et al., 2018; Manem et al., 2018) (Table 1) For instance, a genomic/transcriptomic/proteomic combined approach has confirmed the existence of the known molecular subtypes (LumA, LumB, HER2+, and BL) of breast cancer (Cancer Genome Atlas Network, 2012) as well as allowing identification of novel therapeutic targets in PDX models (Huang K. L. et al., 2017). Recently, a comprehensive analysis of clinical, genomics, and transcriptomics data has uncovered the TNBC landscape (Jiang et al., 2019). Proteogenomics has challenged the way in which somatic mutations contribute to signaling changes (Mertins et al., 2016), highlighting the need of both these analyses to confirm the therapeutic importance of a genetic alteration. For instance, patients lacking HER2 amplification were found to have enriched HER2 signaling (Pierobon et al., 2018), underlining the importance of analyzing changes in signaling to plan the correct therapeutic approach. With the development of single cell analysis in genomics, transcriptomics and proteomics (Linnarsson and Teichmann, 2016; Hong et al., 2019; Marx, 2019; Wagner et al., 2019) there are opportunities to better understand breast cancer heterogeneity and the role of the microenvironment. Finally, it would be fascinating to integrate ‘omics approaches with radiomics (quantitative information from digital images) (Pinker et al., 2018) and with imaging-based mass spectrometry that is rapidly changing the field of spatial proteomics (Keren et al., 2018) to guide patient-specific therapy or patient stratification.

## ‘Omics Approaches Applied to Existing Breast Cancer Models

Integrating ‘omics approaches with traditional methods has already helped underline the validity of some of the models, for example, highlighting that omics profiles are maintained in PDX models through multiple passages (Zhang et al., 2013). Multiomics technologies have also facilitated novel discoveries in existing models (Chakraborty et al., 2018). A combination of genomics, transcriptomics and proteomics has elucidated drivers of mesenchymal-to-epithelial transition in 2D culture (Bhatia et al., 2019). Transcriptomics in GEMM and SMM-derived cell lines allowed identification of differentially regulated genes and their contribution to metastases (Yang et al., 2017) Transcriptomics and proteogenomics in PDXs have finally helped to profile gene/proteins expression to identify novel targets (Huang K. L. et al., 2017).

‘Omics technologies have not only improved the power of traditional models in breast cancer research, but also revolutionized the analysis of patient samples, making them an indispensble tool in translational studies. Integration of ‘omics approaches requires powerful computational and statistical methods to analyze and interpret the vast quantity of available data, for instance combining linear mathematical models with machine learning and network science principles (Manem et al., 2018). This requires collaboration between cancer scientists, computational biologists and medical statisticians to create robust methods to gain insights into cancer biology and to inform clinical trials and personalized therapeutic regimes.

## Conclusion and Perspectives

With ‘omics technologies applied to patient samples becoming robust, our understanding of the mechanisms driving breast cancer and the discovery of novel biomarkers and therapeutic targets have improved significantly over the last few years (Chakraborty et al., 2018; Manem et al., 2018). For instance, the use of molecular assays, including OncotypeDx and MammaPrint in the clinic is based on advancements in genomic technologies (Gupta et al., 2015; Vieira and Schmitt, 2018). Transparent sharing of ‘omics data in databases like COSMIC (Forbes et al., 2008), PRIDE (Jones et al., 2006) and others (Table 2) will allow unbiased analysis of available data by different groups to find previously unnoticed potential genes or proteins of interest as biomarkers or therapeutic targets.

**TABLE 2:** A selection of ‘omics data repositories built for data sharing and to support research questions (Bamford et al., 2004; Fontaine et al., 2011; Omenn, 2014; Speake et al., 2015; Tomczak et al., 2015; Clough and Barrett, 2016; Rudnick et al., 2016; Chou et al., 2019; Tate et al., 2019).

**Database**	**‘Omics data**					**Additional information**	**References**
	**G**	**T**	**P**	**M**	**E**		
Catalogue of Somatic Mutations in Cancer (COSMIC)	×	×			×	COSMIC contains data from over 13 million tumor samples, identifying 6 million coding mutations and over 19 million non-coding mutations. This resource collates all genes implicated in cancer through somatic mutation, of which 719 are currently listed.	Bamford et al., 2004; Tate et al., 2019
The Cancer Genome Atlas (TCGA)	×	×	×		×	TCGA contains multi omic data for 30 different tumor types. In regards to breast cancer it has enabled confirmation of the existence of the four main breast cancer subtypes, it has identified several novel breast cancer drivers and it has identified potentially druggable novel targets.	Tomczak et al., 2015
Clinical Proteomic Tumor Analysis Consortium (CPTAC)			×			CPTAC contains mass spectrometry-based proteomic analysis of tumors from TCGA. The aim of CPTAC is to create a proteogenomic resource where dysregulated proteins and phosphorylation sites can be identified and potentially connected to genomic alterations.	Rudnick et al., 2016
Proteomics Identification Database (PRIDE)			×			PRIDE aims to be a resource for open access sharing of mass spectrometry data, not just across cancer. They currently have over 9200 datasets available, including 297 breast cancer datasets.	Jones et al., 2006
GENIE	×					GENIE combines genomic and clinical data in an attempt to associate genomic alterations with phenotypic changes	Fontaine et al., 2011
GXB		×				GXB compiles immunological transcriptomic data	Speake et al., 2015
Genomic Expression Omnibus (GEO)	×	×			×	GEO is a database of transcriptomic and epigenomic data	Clough and Barrett, 2016
Human Proteome Organization (HUPO)			×			The human proteome project, run by HUPO aims to identify all the proteins in the human proteome and to begin to assess their functionalities and interactions	Omenn, 2014
Transciptome Alterations in Cancer Omnibus (TACCO)		×				TACCO is a resource for identifying differentially regulated transcripts within different cancer types and combining these with survival data to determine prognosis based ongene expression profiles	Chou et al., 2019

*G, genomics; T, transcriptomics; P, proteomics; M, metabolomics; E, epigenomics.*

The implementation of ‘omics approaches in clinical practice will allow analysis of changes in patients at a global level by improving diagnosis and choice of therapeutic plan so far based on a few markers. We predict that ‘omics technologies-guided biomarker identification will allow early tumor detection so that treatments can start earlier and that the identification of novel targets will decrease reliance on non-targeted therapies, thus improving the quality of life for breast cancer patients.

## Author Contributions

All authors listed have made a substantial, direct and intellectual contribution to the work, and approved it for publication.

## Conflict of Interest

The authors declare that the research was conducted in the absence of any commercial or financial relationships that could be construed as a potential conflict of interest.

## Footnotes

1 https://www.cancer.gov/about-nci/organization/ccg/research/structural-genomics/tcga
